# Communicating with Patients about Harms and Risks

**DOI:** 10.1371/journal.pmed.0020042

**Published:** 2005-02-22

**Authors:** Andrew Herxheimer

## Abstract

Health professionals, says Herxheimer, must share their understanding of the benefits and harms of any treatment with patients and their families

Everything that doctors and other health workers do involves communication about the benefits and harms to be expected from interventions—whether they are therapeutic, diagnostic, or prophylactic. As health-care professionals, we need to share our understanding and perceptions of benefits and harms with patients and their families as fully as we can. We also have to share them with other professionals. When we do so we have to remember that how we personally value particular benefits and harms may well differ from how another person values them.

A clinician who recommends an intervention does so in the belief that its benefits outweigh the harms that it can cause. In most consultations there is little time in which to explain in detail what these benefits and harms are, or to find out what the patient thinks about them. Moreover, most clinicians are not trained or practised at describing and explaining benefits and harms clearly to patients, and much of the time they also lack important information about these aspects.

## “Risk” Versus “Harm”

The problems begin with the word “risk”. Very often people use it when they mean “harm”, and this causes ambiguities and confusion. The widely used expression “benefit/risk ratio” is meaningless—no such ratio exists. Before a decision is made to use an intervention, its benefits and harms must be weighed, ideally by the clinician and the patient together. Other advantages and disadvantages, such as convenience and cost, may also be relevant. This analysis requires use of the same dimensions for considering both benefits and harms. These dimensions have not been generally recognised or taught, though they seem obvious enough.[Fig pmed-0020042-g001]

**Figure pmed-0020042-g001:**
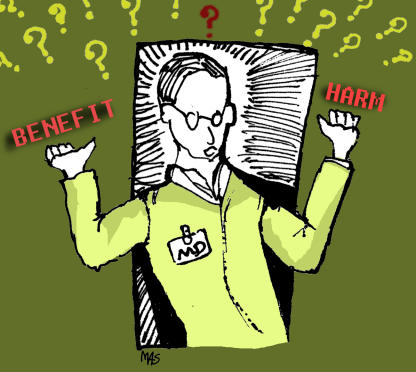
Health professionals need to share their understanding of harms and benefits with patients and their families (Illustration: Margaret Shear, Public Library of Science)

In this context any benefit or harm has four dimensions (see sidebar). The clinician is expected to know or find out about the nature and probability of each benefit and harm, and how to maximise benefits and minimise harms. A great many clinicians do not meet this expectation, and often that is not their fault. But only patients can say how they regard the hoped for benefits and the possible harms, though they may need help to think clearly about them. Clinicians should identify how much the benefits matter to their patient—for example, are the benefits of taking a medicine or having an operation “worth the trouble”?—and whether a specific harm is particularly threatening or would be intolerable to that particular patient. People's fears, wishes, and priorities differ greatly and unpredictably.

The deepening of the voice that occurs with long-term use of tamoxifen for breast cancer, and that is usually irreversible, is an example of a side effect that prescribers, manufacturers, and drug regulators have considered trivial and have largely ignored. While this side effect does not bother most women, for professional or keen amateur singers it is a disaster—it can rob them of what they enjoy most.

A patient who is offered a treatment with serious implications needs time and encouragement to think, and to talk to other people, before making a decision. Three major issues are important in helping patients with decision-making: obtaining reliable information about benefits and harms, effectively communicating probabilities to the patient, and determining what to do to reverse or mitigate harmful effects when they occur.

## Explaining Uncertainties and Probabilities

Innumeracy is very widespread. Many people cannot handle percentages, and most are unclear about the meaning of “relative risk”, “absolute risk”, “odds ratio”, and “number needed to treat”, including many clinicians who want to tell their patients about the likelihood of benefits or harms [1]. Gigerenzer has shown that information on outcomes presented as natural frequencies (for example, “a one in five chance”) is much easier to understand than information expressed in probabilities (for example, “a 20% chance”) [2]. The reason, he suggests, is that “natural frequencies result from natural sampling, the process by which humans and animals have encountered information about risks during most of their evolution” ([1], p. 48–49). So, to put it in its simplest form, it is most effective to say to a patient “this treatment is effective in eight patients out of ten”, or “this drug causes nausea or vomiting in three people out of every ten who use it”.

**The Four Dimensions of Any Benefit or Harm**

1. Its **nature**, described by its quality, its intensity, and its time course (onset, duration, and reversibility).
2. The **probability** that it will occur.
3. Its **importance to the person experiencing it.**
4. How the benefit can be **maximised**, or the harm **prevented or minimised.**

## Obtaining Reliable Information

The effectiveness of an intervention (the extent to which a treatment produces a beneficial effect when implemented under the usual conditions of clinical care for a particular group of patients) is most readily estimated from controlled clinical trials. With the rise of evidence-based medicine, there are now many more critical analyses, systematic reviews, and meta-analyses of the best evidence, as in the rapidly growing Cochrane Database of Systematic Reviews (www.cochrane.org). Nevertheless, there are huge gaps—we still lack reliable evidence about many important interventions.

Even in the case of common conditions for which many high-quality trials have been published, trial reports have not addressed some elementary questions, for example, on optimal drug dosage and duration of treatment. Almost all drug effects are related to dose [3], but we rarely learn what the lowest effective dosage is in different circumstances, and how far it is worth increasing the dosage if the effect is insufficient.

Details of dose–response relationships are hardly ever published. They are usually studied early in the development of a drug in a relatively small number of volunteers, and are used to decide on the dosages to be used in the major clinical trials that will support the licensing application. They are regarded as internal working data of the company, which is not interested in publishing them. Regulatory agencies do not appear to ask for them or examine them critically. Everybody now habitually uses means and group differences to judge effectiveness, although individuals commonly differ greatly in their sensitivity to both beneficial and harmful effects of drugs. This thoughtless reliance on means and group differences, which ignores an important dimension of evidence, is now embedded in “evidence-based practice”. Marketing departments prefer a “one size fits all” approach: it is hard to sell a drug whose dose may need to be titrated.

Another important unanswered question is the variation in response between individuals. Because controlled trials compare treatments they usually report only group means and test their significance. This gives clinicians no help in treating people who are more or less sensitive to the drug than average.

Reliable information on harms is for several reasons even harder to get [4]. Far less research is done to investigate them. Companies do not want to do more work than regulators require, and once they have marketed a drug they hesitate to pay for more research, especially if the results might be inconvenient. Independent public funding hardly exists. Many kinds of harm—often unforeseen and uncommon—need to be first detected and then diligently investigated and analysed. And the available research designs yield less robust evidence than can be obtained for predefined therapeutic effects. Here, too, dose–response data are almost completely lacking.

We still lack reliable evidence about many important interventions.

Thus, much of the time prescribers and patients are poorly informed, and have to rely on cautious exploration, common sense, and personal experience. Nevertheless, as Yoon Kong Loke has pointed out, there are certainly some situations at the bedside when it is particularly important to base treatment decisions on as precise an estimate as possible of the balance of benefits and harms [5]. An example is when there is a narrow margin between benefit and harm, such as giving aspirin to a patient with a stroke who has a past history of gastrointestinal haemorrhage. Another example is when there are several efficacious treatments with differing safety profiles, such as warfarin versus aspirin in a healthy, middle-aged patient with lone atrial fibrillation.

## Checking Effectiveness and Detecting and Dealing with Harms

Doctor and patient need to work together to check that the treatment is as effective as intended, and to detect possible harm promptly. Monitoring can be left to patients if they (or the family) can understand what to watch for and what to do if a problem arises. If not, or if examination or lab tests are necessary, then monitoring and follow-up by a nurse or doctor will need to be arranged.

Here is a checklist of points for clinicians—and of course also drug regulators—to consider. (1) When an adverse effect occurs, should the dose be reduced, or the drug changed? (2) If reduced, by how much? (3) Is reducing the dose possible and practicable with the available preparations? (4) How and over what time should the effect of the change be observed and assessed? (5) Should the patient, as well as the clinician, keep the records of adverse effect(s) and their intensity and timing? Such notes can help both the patient and current and future doctors. Medication experiences can remain relevant for life. (6) Should an adverse effect be reported to a local or national adverse drug reaction register? (If in doubt, the answer is yes).

## Conclusion

Effective communication about harms and risks is an essential component of care, and it requires learning, preparation, and rehearsal. The onus lies with professionals to persuade and to teach patients to play their part in coming to an informed decision about treatments.
